# Clinical Relevance of High Plasma Trough Levels of the Kinase Inhibitors Crizotinib, Alectinib, Osimertinib, Dabrafenib, and Trametinib in NSCLC Patients

**DOI:** 10.1097/FTD.0000000000001120

**Published:** 2023-06-20

**Authors:** Lishi Lin, Hannerieke J. Barkman, Egbert F. Smit, Adrianus J. de Langen, Neeltje Steeghs, Jos H. Beijnen, Alwin D. R. Huitema

**Affiliations:** * Department of Pharmacy and Pharmacology, The Netherlands Cancer Institute-Antoni van Leeuwenhoek Hospital, Amsterdam, The Netherlands;; † Department of Thoracic Oncology, Netherlands Cancer Institute-Antoni van Leeuwenhoek, Amsterdam, The Netherlands;; ‡ Department of Pulmonology, Leiden University Medical Center, Leiden, The Netherlands;; § Department of Medical Oncology, Netherlands Cancer Institute-Antoni van Leeuwenhoek, Amsterdam, The Netherlands;; ¶ Department of Pharmaceutical Sciences, Utrecht University, Utrecht, The Netherlands;; ║ Department of Pharmacology, Princess Máxima Center for Pediatric Oncology, Utrecht, The Netherlands; and; ** Department of Clinical Pharmacy, University Medical Center Utrecht, Utrecht University, Utrecht, The Netherlands.

**Keywords:** non–small-cell lung cancer, kinase inhibitors, toxicity, adverse events, therapeutic drug monitoring

## Abstract

**Background:**

the study aims to evaluate whether high plasma trough levels of the kinase inhibitors (K.I.s) crizotinib, alectinib, osimertinib, dabrafenib, and trametinib were associated with a higher risk of toxicity in non–small-cell lung cancer patients.

**Methods:**

In this retrospective cohort study, patients with non–small-cell lung cancer treated with the selected K.I.s were included if at least one plasma trough level at steady state (C_min,ss_) was available. Data were extracted from electronic medical records and laboratory databases. The high group for each K.I. was defined as 10% of patients with the highest first C_min,ss_. The remaining patients were placed in the non-high group. The frequency of dose-limiting toxicities (DLTs), defined as adverse events leading to dose reduction, dose interruption, or permanent discontinuation, was compared between the 2 groups.

**Results:**

A total of 542 patients were included in the different K.I. groups. A high C_min,ss_ of crizotinib (n = 96), alectinib (n = 105), osimertinib (n = 227), dabrafenib (n = 52), and trametinib (n = 62) correlated with a C_min,ss_ ≥490, ≥870, ≥405, ≥150, and ≥25 ng/mL, respectively. DLTs were more common in the alectinib high group than in the alectinib non-high group (64% vs. 29%, *P* = 0.036). Liver toxicity was observed in 4 (36%) patients in the high group and 5 (5%) patients in the non-high group (*P* = 0.007). For other K.I.s, no significant differences were observed in the frequency of DLTs between the high and non-high groups.

**Conclusions:**

For alectinib, high C_min,ss_ was correlated with a higher risk of DLT. No differences in the frequency of DLTs were observed between the high and non-high groups for crizotinib, osimertinib, dabrafenib, and trametinib.

## INTRODUCTION

Lung cancer is one of the most prevalent types of cancer worldwide, with approximately 85% of cases being non–small cell lung cancer (NSCLC).^1^ In the last 2 decades, the discovery of molecular alterations driving tumor initiation and progression has led to the approval of kinase inhibitors (K.I.s) that target oncogenic driver mutations. The most frequent oncogenic drivers in NSCLC are the Kirsten rat sarcoma viral oncogene (KRAS), epidermal growth factor receptor, and anaplastic lymphoma kinase, whereas less common oncogenic driver aberrations involve ROS1, MET, and BRAF.^2,3^

In treating NSCLC, ROS1, and anaplastic lymphoma kinase fusions and epidermal growth factor receptor and BRAF mutations are targeted, among others, with crizotinib, alectinib, osimertinib, and a combination of dabrafenib and trametinib, respectively. For all these K.I.s, fixed dosing is the standard. However, the pharmacokinetic exposure to K.I.s is highly variable, whereas many K.I.s show an exposure–response relationship for efficacy and toxicity.^4^ The current research aimed at optimizing treatment outcomes by therapeutic drug monitoring mainly focuses on low plasma concentrations to improve efficacy.^5^ In contrast to low plasma concentrations, high plasma concentrations can potentially lead to an increased risk of toxicity. Because only limited data exist on the exposure–toxicity relationship of K.I.s, it is unclear whether high plasma concentrations observed in daily clinical practice are associated with a higher risk of toxicity. Therefore, the aim of this study was to evaluate whether high plasma trough levels of crizotinib, alectinib, osimertinib, dabrafenib, and trametinib are associated with a higher risk of toxicity in NSCLC patients.

## MATERIALS AND METHODS

This retrospective observational cohort study was conducted at the Netherlands Cancer Institute—Antoni van Leeuwenhoek hospital (NKI-AvL), Amsterdam, the Netherlands. NSCLC patients who were treated with crizotinib, alectinib, osimertinib, dabrafenib, or trametinib (irrespective of the dose and frequency of administration) between September 2012 and December 2020 with at least one trough level at steady state (C_min,ss_) were included in this study. Data-cutoff was August 19, 2021. At the NKI-AvL, plasma levels of the studied K.I.s were collected as part of the standard of care during follow-up visits to the outpatient clinic. Plasma levels were measured using validated liquid chromatography with tandem mass spectrometry detection.^6,7^ The last drug intake and plasma sampling date and time were used to calculate the trough levels. Log-linear extrapolation was used to determine trough levels of crizotinib, alectinib, osimertinib, and trametinib, whereas, for dabrafenib, a population pharmacokinetic model was used.^8^ Plasma samples collected within half an hour after the last drug intake were interpreted as trough levels. The therapeutic target trough levels for crizotinib, alectinib, osimertinib, dabrafenib, and trametinib were ≥235, ≥435, ≥166, ≥46.6, ≥10.6 ng/mL, respectively.^4,9^

For each K.I. group, patients were divided into 2 groups based on the first C_min,ss_ measured. High exposure to K.I.s was defined as patients with the 10% highest C_min,ss_ determined for a certain K.I., because there is no clear definition of “high exposure” yet and for statistical analysis. The remaining patients were assigned to the non-high group. Patient characteristics, medication use, and the occurrence of dose-limiting toxicities (DLTs) were extracted from electronic medical records (EMR), whereas data on plasma levels were extracted from the laboratory databases. The investigational review board of the NKI-AvL approved this observational cohort study, and the need for written informed consent was waived.

The primary endpoint were DLTs, defined as adverse events leading to dose reduction, dose interruption, or permanent treatment discontinuation. A distinction was made in the DLTs that occurred before and after the first C_min,ss_. Only the DLTs after the first C_min,ss_ were used in the correlation with the first C_min,ss,_ because the trough level is not representative of the DLTs before dose reductions. In addition, for each K.I., the treatment duration for the high and non-high K.I. groups was determined.

The Fisher exact test was used for categorical data, and the two-sample *t* test or Mann–Whitney test was used for continuous outcome variables, depending on the appropriateness of the tests. The Kaplan–Meier method was used to determine the median treatment duration of the groups. A *P*-value <0.05 was regarded as statistically significant in all cases. All statistical analyses were performed using R version 4.1.1 (R Foundation for Statistical Computing, Vienna, Austria).

## RESULTS

### Characteristics of the Study Population

A total of 542 patients were included in this study. The patient characteristics for each K.I. group are depicted in Table 1. Table 2 depicts the starting dose of the K.I.s and the dose at the time of the first trough level. In this study, high trough levels of crizotinib, alectinib, osimertinib, dabrafenib, and trametinib correlated with trough levels ≥490, ≥870, ≥405, ≥150, and ≥25 ng/mL, respectively. For some K.I.s, a difference in patient characteristics was observed between the non-high and high groups. For crizotinib, significant differences were observed in age, weight, and height. For alectinib, patients in the high group had a lower body weight, whereas, for osimertinib, brain metastases and worse performance status were observed more often in the high group. No statistically significant differences were observed between the high and non-high groups for dabrafenib and trametinib.

**TABLE 1.** Patient Characteristics in the Non-high and High Group for Crizotinib, Alectinib, Osimertinib, Dabrafenib, and Trametinib at Baseline

	Crizotinib			Alectinib			Osimertinib			Dabrafenib			Trametinib		
	Non-high, n = 86	High, n = 10	*P*	Non-high, n = 94	High, n = 11	*P*	Non-high, n = 204	High, n = 23	*P*	Non-high, n = 47	High, n = 5	*P*	Non-high, n = 56	High, n = 6	*P*
Sex, female (%)	39 (45)	7 (70)	0.187	48 (51)	7 (64)	0.531	152 (75)	19 (83)	0.457	28 (60)	3 (60)	1	33 (59)	4 (67)	1
Age (yr), mean (SD)	58 (14)	66 (6)	0.005	58 (13)	61 (17)	0.583	65 (11)	61 (9)	0.123	68 (9)	70 (10)	0.621	67 (10)	69 (7)	0.446
Stage (%)			0.595			0.494			1			N.A.			N.A.
III	8 (9)	—		5 (5)	1 (9)		3 (1)	—		—	—		—	—	
IV	78 (91)	10 (100)		89 (95)	10 (91)		201 (99)	23 (100)		47 (100)	5 (100)		56 (100)	6 (100)	
Brain metastasis (%)	12 (14)	4 (40)	0.059	26 (28)	5 (46)	0.295	68 (33)	14 (61)	0.012	7 (15)	1 (20)	0.1	7 (13)	1 (17)	0.580
ECOG PS (%)			0.846			0.791			0.006			0.381			0.211
0	37 (43)	4 (40)		30 (32)	3 (27)		74 (36)	3 (13)		7 (15)	2 (40)		9 (16)	2 (33)	
1	40 (47)	5 (50)		46 (49)	5 (46)		103 (51)	11 (48)		28 (57)	2 (40)		32 (57)	2 (33)	
2	5 (6)	1 (10)		6 (6)	1 (9)		21 (10)	6 (26)		7 (15)	—		5 (9)	2 (33)	
3 of 4	—	—		3 (3)	—		5 (3)	3 (13)		1 (2)	—		1 (2)	—	
NA	4 (5)	—		9 (10)	2 (18)		1 (0.5)	—		5 (11)	1 (20)		9 (16)	—	
Length (cm), mean (SD)	175 (11)	164 (11)	0.015	174 (10)	167 (13)	0.105	169 (10)	170 (10)	0.775	168 (12)	167 (11)	0.871	170 (11)	164 (9)	0.151
Body weight (kg), mean (SD)	78 (15)	64 (10)	0.005	80 (15)	70 (10)	0.012	72 (15)	72 (16)	0.921	72 (14)	81 (18)	0.363	76 (24)	63 (10)	0.077

**TABLE 2.** The Drug Dose of Crizotinib, Alectinib, Osimertinib, Dabrafenib, and Trametinib at the Time of Treatment Initiation and the Time of First Trough Level at Steady State (C_min,ss_) Determination

	Starting Dose, n (%)		Dose at First C_min_, n(%)	
	Non-high	High	Non-high	High
Crizotinib (n = 96)				
250 mg b.i.d.	75 (87)	10 (100)	61 (71)	9 (90)
200 mg b.i.d.	5 (6)	—	11 (13)	1 (10)
250 mg q.d.	5 (6)	—	12 (14)	—
200 mg q.d.	1 (1)	—	2 (2)	—
Alectinib (n = 105)				
600 mg b.i.d.	85 (91)	11 (100)	67 (71)	11 (100)
450 mg b.i.d.	5 (5)	—	21 (22)	—
300 mg b.i.d.	4 (4)	—	6 (6)	—
Osimertinib (n = 227)				
80 mg q.d.	191 (94)	20 (87)	187 (92)	19 (83)
160 mg q.d	10 (5)	3 (13)	6 (3)	4 (17)
120 mg q.d.	—	—	1 (0.5)	—
40 mg q.d.	3 (1)	—	10 (5)	—
Dabrafenib (n = 52)				
150 mg b.i.d.	45 (96)	5 (100)	35 (74)	5 (100)
100 mg b.i.d.	—	—	2 (4)	—
75 mg b.i.d.	2 (4)	—	7 (15)	—
50 mg b.i.d.	—	—	3 (6)	—
Trametinib (n = 62)				
2 mg q.d.	53 (95)	6 (100)	43 (77)	6 (100)
1.5 mg q.d.	—	—	4 (7)	—
1 mg q.d.	2 (4)	—	6 (11)	—
0.5 mg q.d.	1 (2)	—	3 (5)	—

B.i.d, twice a day; q.d., once daily.

### Dose-Limiting Toxicities

**FIGURE 1.**
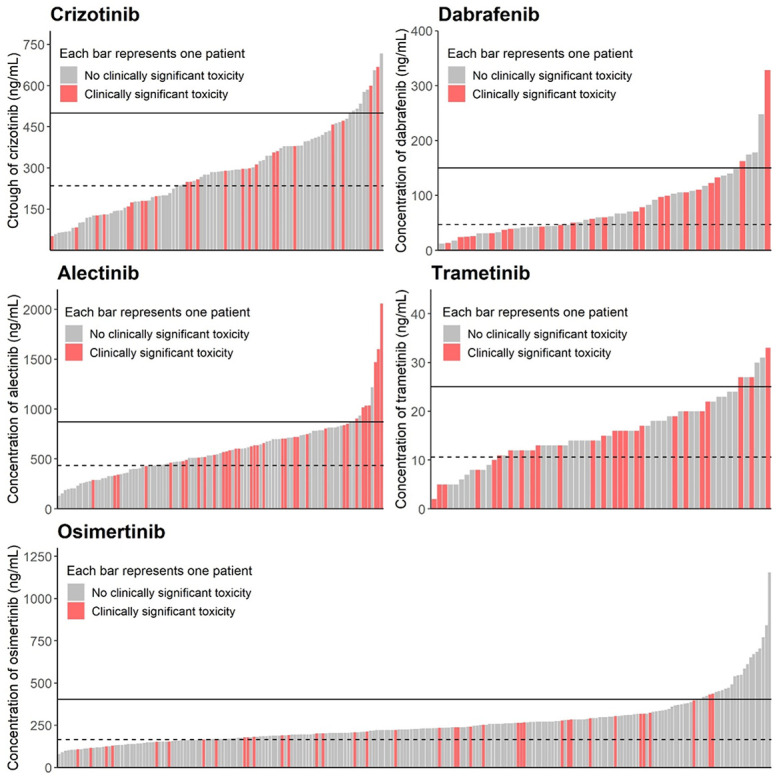
The first trough level of all patients and the occurrence of dose-limiting toxicities for each kinase inhibitor (K.I). The non-high and the high group are separated by the horizontal line, whereas the dotted line represents the therapeutic target trough level for each K.I., according to.^4,9^

The first C_min,ss_ of all patients and the occurrence of DLTs after C_min,ss_ are depicted in Figure 1. During treatment with crizotinib, alectinib, osimertinib, dabrafenib, and trametinib, 37 (39%), 51 (49%), 50 (22%), 30 (57%), and 34 (55%) patients experienced DLTs, respectively. Table 3 depicts the frequency of all DLTs during treatment, before, and after the first C_min,ss_ for high and non-high K.I. groups. The median time from treatment initiation to the first C_min,ss_ with an interquartile range was 78 (40–152), 43 (24–84), 52 (29–99), 48 (26–126), and 54 (38–133) days for crizotinib, alectinib, osimertinib, dabrafenib, and trametinib, respectively.

**TABLE 3.** Frequency of Total Dose-Limiting Toxicities (DLT) in the Non-high and High Group for Crizotinib, Alectinib, Osimertinib, Dabrafenib, and Trametinib before and after the First Trough Level at Steady State (C_min,ss_)

	Crizotinib		Alectinib		Osimertinib		Dabrafenib		Trametinib	
	Non-high, n = 86	High, n = 10	Non-high, n = 94	High, n = 11	Non-high, n = 204	High, n = 23	Non-high, n = 47	High, n = 5	Non-high, n = 56	High, n = 6
Total DLT (%)	34 (40)	3 (30)	44 (47)	7 (64)	47 (23)	3 (13)	27 (57)	2 (40)	31 (55)	3 (50)
DLT because of symptoms (%)	17 (20)	2 (20)	31 (33)	2 (18)	41 (20)	2 (9)	20 (43)	2 (40)	23 (41)	1 (17)
DLT because of lab values (%)	12 (14)	1 (10)	8 (9)	3 (27)	6 (3)	1 (4)	4 (9)	—	4 (7)	—
DLT because of combination of symptoms and lab values (%)	5 (6)	—	5 (5)	2 (18)	—	—	3 (6)	—	4 (7)	2 (33)
										
DLTs before C_min,ss_ (%)	15 (17)	1 (10)	22 (23)	—	12 (6)	1 (4)	12 (26)	—	15 (27)	—
Dose reduction before C_min,ss_ (%)	11 (13)	1 (10)	15 (16)	—	9 (4)	—	11 (23)	—	11 (20)	—
Dose interruption before C_min,ss_ (%)	10 (13)	—	12 (13)	—	6 (3)	1 (4)	7 (15)	—	10 (18)	—
										
DLTs after C_min,ss_ (%)	21 (24)	2 (20)	28 (30)	7 (64)	35 (17)	2 (9)	20 (43)	2 (40)	22 (39)	3 (50)
Dose reduction after C_min,ss_ (%)	15 (17)	1 (10)	22 (23)	5 (45)	26 (13)	2 (9)	9 (19)	1 (20)	7 (13)	2 (33)
Dose interruption after C_min,ss_ (%)	10 (12)	—	15 (16)	3 (27)	13 (6)	—	18 (38)	—	17 (30)	2 (33)
Discontinuation after C_min,ss_ (%)	7 (8)	1 (10)	6 (6)	2 (18)	4 (2)	—	4 (9)	1 (20)	8 (14)	2 (33)

For alectinib, patients in the high group experienced more DLTs after the first C_min,ss_ compared with those in the non-high group, 64% and 30% (*P* = 0.039), respectively. There was also a difference in the type of toxicity leading to DLTs, because liver toxicity was observed in 4 (36%) patients in the high group and 5 (5%) patients in the non-high group (*P* = 0.007). For the other K.I.s, no significant differences were observed in the frequency of DLTs between the high and non-high groups or the type of toxicity leading to DLTs.

In the crizotinib high group, treatment was discontinued in one patient because of pneumonitis, whereas in the alectinib high group, treatment was discontinued in 2 patients because of liver toxicity. In the osimertinib high group, no treatment discontinuation occurred because of toxicity. In the dabrafenib high group, treatment was discontinued in one patient because of pneumonitis. For the trametinib high group, treatment was discontinued in 2 patients because of nephrotoxicity and benign paroxysmal positional vertigo, for which initially, a dose interruption was planned, but treatment was not reinitiated because of progression.

### Treatment Duration

**FIGURE 2.**
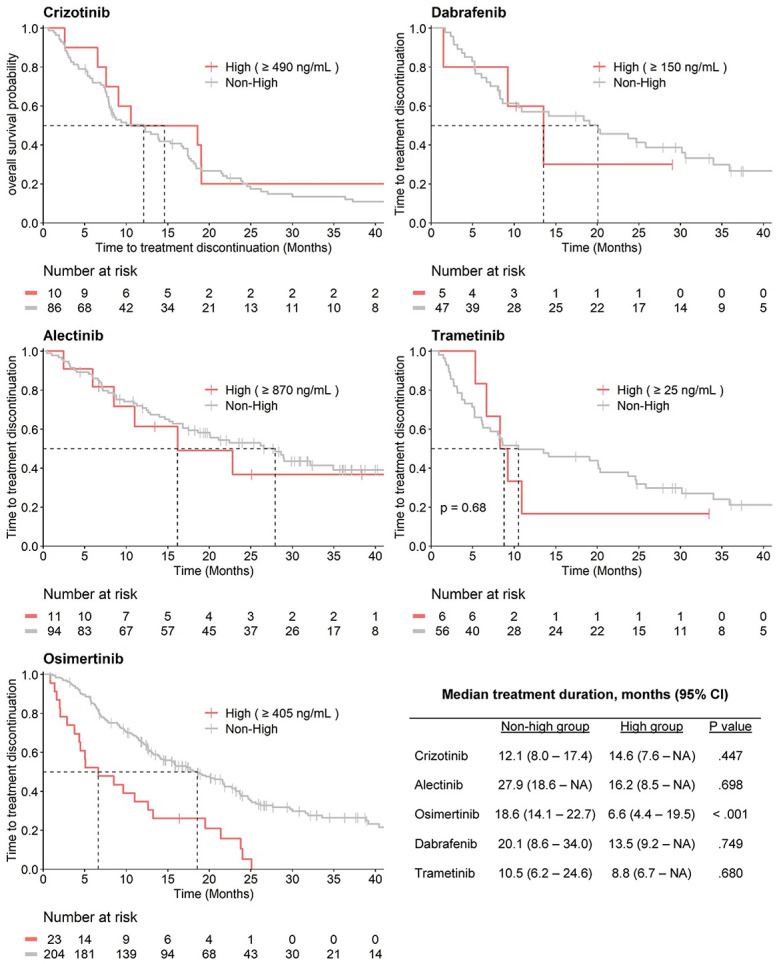
The median treatment duration of the non-high and high groups for crizotinib, alectinib, osimertinib, dabrafenib, and trametinib.

The Kaplan–Meier curves for the high and non-high groups are shown in Figure 2. For osimertinib, a statistically significant shorter median treatment duration was observed for patients in the high group compared with the non-high group, respectively 6.6 versus 18.6 months (*P* < 0.001), consistent with the unfavorable patient characteristics in the high group. For the other 4 K.I.s, no significant differences were observed in treatment duration.

## DISCUSSION

In this observational cohort study, the clinical relevance of high trough levels of crizotinib, alectinib, osimertinib, dabrafenib, and trametinib in the context of toxicity in daily clinical practice was studied. A high trough level of the K.I.s corresponded to ≥490, ≥870, ≥405, ≥150, and ≥25 ng/mL, respectively. These trough levels were roughly twice as high as the target trough levels. Alectinib was the only K.I. for which a high C_min,ss_ was correlated with more DLTs, which often expressed itself in the form of liver toxicity. For the other K.I.s, no correlation was observed between high C_min,ss,_ and DLTs.

A study investigating the exposure–toxicity relationship of alectinib from the final pooled phase II data revealed no relationship between alectinib exposure and the incidence of serious adverse event.^10^ In contrast, our study showed that there probably is an upper limit to the therapeutic window of alectinib above which DLTs occur more frequently. Liver toxicity was observed strikingly more frequently in the high group. Similarly, in the phase II studies, the most common adverse events leading to dose reduction or interruption were increased blood bilirubin and alanine aminotransferase.^11^

For osimertinib, a linear relationship has been observed between exposure and rash, diarrhea, and QTc interval.^12^ Despite this exposure–toxicity relationship, patients with high osimertinib trough levels in our study did not experience more DLTs than those with lower trough levels. No significant exposure–toxicity relationship was observed for crizotinib in a study using data from 2 pivotal trials.^13^ For dabrafenib, 2 studies showed an exposure–toxicity relationship in melanoma patients, in contrast to another study in which this relationship was not found. For trametinib, no exposure–toxicity relationship was observed in these studies.^14–16^ Therefore, our study's results of crizotinib, dabrafenib, and trametinib are in line with the literature.

Except for osimertinib, the treatment durations did not differ between patients in the high and non-high groups, suggesting that patients with higher trough levels did not experience more toxicity, leading to shorter treatment durations. For osimertinib, patients with higher trough levels had shorter treatment duration. Because patients in the high group had more frequent brain metastases and worse performance status at the start of treatment, the worse health state is expected to be the reason for faster progression.

One of the strengths of this study is the use of data from daily clinical practice in which plasma levels were collected as part of the standard of care, making this a valuable addition to the data from strongly preselected patient populations in pivotal trials. A limitation of this study was that the data were collected retrospectively, making us dependent on the information captured in the EMR of patients and the laboratory database. However, this limitation has been partially overcome using DLTs as the primary endpoint, because DLTs are adverse events that are clinically relevant in daily practice and are well documented in the EMR, and can be verified using data on drug dispensing from the hospital pharmacy. Another limitation is the use of only the first trough level to divide the patients into high and non-high groups, because the trough level or possibly the AUC at the time of experiencing DLTs would be of interest. Nonetheless, using the first trough level could also be considered a strength of this study because it can be helpful in the early prediction of an increased risk of DLT in the subsequent course of treatment, as was the case for alectinib in our study. In addition, the number of patients included in the dabrafenib and trametinib groups was relatively small; therefore, the power to find an association between high trough levels and DLTs was limited.

Because the patients in the high K.I. groups had trough levels far beyond the therapeutic target trough level, it may be rational to reduce the dose if patients experience adverse events to improve their quality of life. In addition, in the context of the effective usage of expensive cancer drugs, it can be questioned whether it is rational to have trough levels twice as high as the therapeutic target trough level. Therefore, it would be interesting to study whether these patients could be treated with a lower K.I. dose or with a longer administration interval while the effectiveness is maintained.

## CONCLUSION

In conclusion, a high trough level of alectinib was correlated with a higher risk of DLT, whereas no differences in the frequency of DLTs were observed between the high and non-high groups for crizotinib, osimertinib, dabrafenib, and trametinib.
